# Upregulation of long noncoding RNA TUG1 promotes cervical cancer cell proliferation and migration

**DOI:** 10.1002/cam4.994

**Published:** 2017-01-15

**Authors:** Yingying Hu, Xiangwei Sun, Chenchen Mao, Gangqiang Guo, Sisi Ye, Jianfeng Xu, Ruanmin Zou, Jun Chen, Ledan Wang, Ping Duan, Xiangyang Xue

**Affiliations:** ^1^ Department of Obstetrics and GynecologySecond Affiliated HospitalWenzhou Medical UniversityWenzhouZhejiang325000China; ^2^ Department of General SurgeryFirst Affiliated HospitalWenzhou Medical UniversityWenzhouZhejiang325000China; ^3^ Department of Microbiology and ImmunologyInstitute of Molecular Virology and ImmunologyInstitute of Tropical MedicineWenzhou Medical UniversityWenzhouZhejiang325000China; ^4^ Department of Obstetrics and GynecologyFirst Affiliated HospitalWenzhou Medical UniversityWenzhouZhejiang325000China

**Keywords:** Bcl‐2, cervical cancer, epithelial–mesenchymal transition, LncRNA, TUG1

## Abstract

Long noncoding RNAs (lncRNAs), a novel class of transcripts that have critical roles in carcinogenesis and progression, have emerged as important gene expression modulators. Recent evidence indicates that lncRNA taurine‐upregulated gene 1 (TUG1) functions as an oncogene in numerous types of human cancers. However, its function in the development of cervical cancer remains unknown. The aim of this research was to investigate the clinical significance and biological functions of TUG1 in cervical cancer. TUG1 was found to be significantly upregulated in cervical cancer tissues and four cervical cancer cell lines by quantitative real‐time polymerase chain reaction (qRT‐PCR). Elevated TUG1 expression was correlated with larger tumor size, advanced international federation of gynecology and obstetrics (FIGO) stage, poor differentiation, and lymph node metastasis. Furthermore, knockdown of TUG1 suppressed cell proliferation with activation of apoptosis, in part by regulating the expression of Bcl‐2 and caspase‐3. Silencing of TUG1 inhibited cell migration and invasion via the progression of epithelial–mesenchymal transition (EMT). Taken together, our findings indicate that TUG1 acts as an oncogene in cervical cancer and may represent a novel therapeutic target.

## Introduction

Cervical cancer is the third most common gynecological malignancy and the fourth most prominent cause of cancer‐related mortality in women worldwide, with a global incidence of 530,000 cases and nearly 275,000 deaths per year 1. Owing to advances in cervical cytology and cervical pathological biopsy, early‐stage and locally advanced cancers may be diagnosed by better testing; hence, the prognosis of cervical cancer has improved significantly in recent years 2, 3. However, the molecular mechanisms underlying the progression and development of this disease remain poorly understood. As a consequence, cervical cancer remains a significant threat to women's health.

With the advent of genome‐wide sequencing analyses, emerging evidence has indicated that the vast majority of noncoding (nc) RNA in the mammalian genome is widely transcribed 4, 5, 6; these ncRNAs are thought to play significant regulatory roles in the initiation of tumorigenesis. Long noncoding RNAs (lncRNAs), at least 200 nucleotides in length, transcribed by RNA polymerase II 5, are important new members of the ncRNA family, yielding little or no protein products. LncRNAs are evolutionarily conserved and numerous reports have demonstrated that they exert an important role in cell proliferation, differentiation, and immune response 7, 8. These responses occur at different molecular levels, such as chromatin modification 9, DNA methylation 10, gene imprinting 11, RNA processing 12, and epigenetic regulation 13. LncRNAs have been highlighted as key components of the cancer transcriptome; therefore, it is reasonable to speculate that, depending on the circumstances, they may function as oncogenes, tumor suppressors, or both. With the exception of lncRNAs identified in preliminary cervical cancer studies, such as HOTAIR 2, GAS5 14, MALAT1 15, MEG3 16, and CCHE1 17, the overall pathophysiological contributions and the underlying molecular mechanisms of specific lncRNAs remain largely unknown.

Taurine‐upregulated gene 1 (TUG1, also known as TI‐227H, Linc00080, and ncRNA00080), a 7.1‐kb gene located at chr22q12.2, consists of four exons. TUG1 was first identified in a microarray screen as a transcript that is overexpressed in taurine‐treated retinal cells 18. The dysregulation of TUG1 is known to affect genes related to the growth and migration of several types of cancers, such as human nonsmall cell lung cancer 19, osteosarcoma 20, hepatocellular carcinoma 21, esophageal squamous cell carcinoma 22, and colorectal cancer 23. However, the relationship between TUG1 expression level and progression of cervical cancer remains poorly documented. Therefore, the main objective of this study was to investigate the clinical significance and biological functions of TUG1 in cervical cancer.

Herein, we report that TUG1 was significantly overexpressed in cervical cancer and it was associated with larger tumor size, advanced international federation of gynecology and obstetrics (FIGO) stage 24, poor differentiation, and lymph node metastasis. In addition, we demonstrated that TUG1 promoted cell proliferation and inhibited cell apoptosis via the regulation of multiple apoptosis‐related proteins. TUG1 was additionally found to enhance migration and invasion by regulating epithelial–mesenchymal transition (EMT) in cervical cancer. Therefore, the results indicated that TUG1 functions as an important oncogene in cervical cancer.

## Materials and Methods

### Patients and specimens

A total of 80 patients were recruited at the Second Affiliated Hospital of Wenzhou Medical University (Zhejiang, China) between March 2013 and March 2014. Cervical tumor samples (*n* = 40) were collected from patients undergoing hysterectomy without radiotherapy or chemotherapy. Cervical intraepithelial neoplasia (CIN) samples were obtained from patients undergoing loop electrosurgical excision procedure (LEEP) and divided into CIN I (*n* = 8), CIN II (*n* = 7), and CIN III (*n* = 6) according to histopathological diagnosis. Normal specimens (*n* = 19) were obtained from patients undergoing surgery because of myoma or adenomyoma. All the normal controls were nonmalignant and were negative for human papilloma virus (HPV) and thinprep cytologic test (TCT). All fresh specimens were snap frozen in liquid nitrogen immediately and stored at −80°C until use.

### Ethical approval

Informed consent was obtained from all individual participants included in the study. All procedures involving human participants were in accordance with the ethical standards of the Human Research Ethics Committee at the Second Affiliated Hospital of Wenzhou Medical University and the 1964 Helsinki declaration and its latest amendments or comparable ethical standards.

### Cell culture

Cervical cancer cells (HeLa, CaSki, SiHa, and C33A) were purchased from the Type Culture Collection of the Chinese Academy of Sciences (Shanghai Institute of Biochemistry and Cell Biology, Shanghai, China). HeLa and CaSki cell lines were grown in RPMI 1640 medium (Gibco, Thermo Scientific, Waltham, MA) containing 10% fetal bovine serum (FBS; Gibco). SiHa and C33A cells were cultured in Dulbecco's modified Eagle medium (Gibco) supplemented with 10% FBS. All cells were grown at 37°C in a cell incubator with a humidified atmosphere containing 5% CO_2_.

### RNA isolation and quantitative real‐time polymerase chain reaction (qRT‐PCR)

Total RNA was isolated from cervical cancer samples and cervical cancer cell lines using TRIzol™ reagent (Invitrogen, Carlsbad, CA) according to the manufacturer's instructions. RNA concentration was determined using a UV‐visible spectrophotometer (NanoDrop™ ND‐1000). Total RNA (1 *μ*g) was used for reverse transcription using the ReverTra Ace® qPCR RT kit (Toyobo, Osaka, Japan). qRT‐PCR was performed using the RNA‐direct™ SYBR® Green Real‐time PCR Master Mix (Toyobo). Primers for TUG1 amplification were as follows: forward: 5′‐GAACTACTGCGGAACCTCAA‐3′; reverse: 5′‐ACTTGGTGAGCACCACTCC‐3′. Glyceraldehyde 3‐phosphate dehydrogenase (GAPDH) was used as an internal standard with forward primer: 5′‐CTGCCAACGTGTCAGTGGTG‐3′; reverse primer: 5′‐TCAGTGTAGCCCAGGATGCC‐3′. All experiments were performed in triplicate. All assays were performed using the ABI 7900HT qRT‐PCR system (Applied Biosystems, Foster City, CA). Relative expression was calculated using the 2^−ΔΔCt^ method 25.

### Transfection

Well‐differentiated cervical carcinoma cells were grown in 6‐well plates and cultured to 60%–70% confluence. Cells were transfected with a mixture of siTUG1 (100 nmol/L) and Lipofectamine® 2000 (Invitrogen) following the manufacturer's protocol. After 6 h, the mixture in Opti‐MEM (Thermo Fisher Scientific) medium was replaced with complete medium. After 48 h of incubation, the transfected cells were used for further experiments. The sequences of siTUG1 were as follows: siTUG1 1#, sense: 5′‐GGGAUAUAGCCAGAGAACAAUUCUA‐3′; antisense: 5′‐UAGAAUUGUUCUCUGGCUAUAUCCC‐3′; siTUG1 2#, sense: 5′‐GCUUGGCUUCUAUUCUGAAUCCUUU‐3′; antisense: 5′‐AAAGGAUUCAGAAUAGAAGCCAAGC‐3′ (GenePharma, Shanghai, China). For inhibitory experiments, 50 *μ*mol/L z‐VAD‐FMK (ZVAD; MedChemExpress, USA) was added to the culture medium after siRNA transfection.

### Cell viability

Cell viability was assessed using the Cell Counting Kit‐8 (CCK‐8) assay (Dojindo, Kumamoto, Japan). The four cervical cancer cell lines (SiHa, CaSki, HeLa, and C33A) were transfected with siTUG1 or negative control siRNA and seeded in 96‐well plates (5 × 10^3^cells/well). At 0, 24, 48, and 72 h, CCK‐8 reagent was added, and the cells were cultured for a further 1–4 h at 37°C. Absorbance at 450 nm was measured using a microplate reader. All experiments were performed in triplicate.

### Analysis of apoptosis by flow cytometry

Cells transfected with siTUG1 for 48 h were resuspended at a concentration of 1 × 10^6^cells/mL. After double staining with 5 *μ*L of propidium iodide (PI) and 5 *μ*L of Annexin V‐FITC from the FITC‐Annexin V Apoptosis Detection Kit (BD Biosciences, San Diego, USA), cells were analyzed with a BD FACS Calibur™ flow cytometer within 1 h. Data analysis was performed using FlowJo (Tree Star, San Carlos, CA).

### Cell colony‐forming assay

Transfected cervical cancer cells (SiHa and HeLa) were planted in 24‐well plates (400 cells/well) and cultured for an additional 2 weeks. Cells were then fixed with 4% paraformaldehyde and stained with 0.5% crystal violet. Colonies containing at least 50 cells were counted under a microscope. CaSki cells transfected with siTUG1 were seeded in 24‐well plates (1000 cells per well) and cultured for an additional week. The assay was performed three times for each treatment.

### Wound healing assay

Transfected cervical cancer cells were grown in 6‐well plates until they attained 90%–95% confluence. Then, the cell monolayer was scratched using a sterile 200‐*μ*L pipette tip to form an artificial wound and cell debris was removed by washing with phosphate‐buffered saline (PBS; Gibco). Cells were further cultured in medium with 2% FBS. Representative images were captured at the designated times, and wound distance was measured. All experiments were performed in triplicate.

### Cell migration and invasion assays

For the cell invasion assay, 24‐well Transwell™ plates with 8.0‐*μ*m‐pore Matrigel™‐coated membranes were used (Corning, NY). Cells (2 × 10^5^) were seeded into the upper chambers with serum‐free medium. The lower chambers were filled with medium containing 20% FBS. After 24 h of incubation, cells were fixed using cold methanol and stained with crystal violet. All migrating or invading cells were counted in five random fields under a microscope. The migration assay was performed similarly without coating the membranes with Matrigel™. All experiments were performed in triplicate.

### Western blot analysis

Total protein was obtained from cells using RIPA lysis buffer (Beyotime, Haimen, China) containing 1% protease inhibitor Cocktail (Sigma‐Aldrich, St. Louis, MO). Equal quantities of protein were separated on a sodium dodecyl sulfate‐polyacrylamide gel and transferred onto a polyvinylidene fluoride membrane (Bio‐Rad, Hercules, CA). The membrane was blocked at room temperature for 2 h with 5% skimmed milk in Tris‐buffered saline with Tween 20, and then probed overnight at 4°C with primary antibodies against Bcl‐2, caspase‐3, cleaved caspase‐3, GAPDH (dilution 1: 1000; Cell Signaling Technology, Danvers, MA), fibronectin, vimentin (1: 200; Santa Cruz Biotechnology, Santa Cruz, CA), or cytokeratin (1: 1000; Dako, Carpinteria, CA). Subsequently, the membranes were exposed to horseradish peroxidase‐labeled IgG for 2 h, and the bands were visualized using a Bio‐Rad imaging system.

### Statistical analyses

All statistical analyses were performed using the SPSS 17.0 software package (IBM, Chicago, IL). Data are reported as means ± standard error of the mean (SEM). Analysis of variance and independent‐sample *t* tests were performed to assess differences between groups. A *P* value of less than 0.05 was considered to represent statistical significance.

## Results

### TUG1 expression is significantly upregulated in cervical cancer

**Figure 1:**
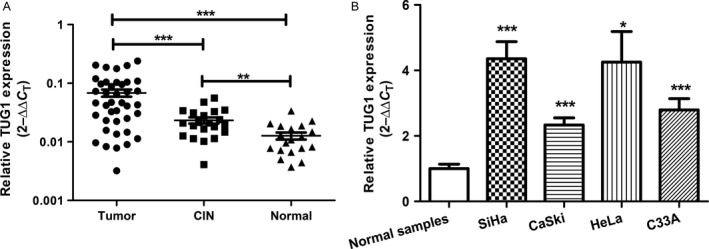
TUG1 is upregulated in cervical cancer tissues and cell lines. (A) The relative expression of TUG1 was increased in cervical cancer and cervical intraepithelial neoplasia (CIN) tissues, as determined by quantitative real‐time polymerase chain reaction (qRT‐PCR) normalized against unpaired normal samples. (B) Expression of TUG1 in SiHa, CaSki, HeLa, and C33A cells was measured and normalized against that of unpaired normal samples using qRT‐PCR. All experiments were performed in triplicate. Data are presented as means ± SEM. *P <* *0.05, **P <* *0.01, ***P <* *0.001.

To determine the expression level of TUG1 in 40 cervical cancer cases, 21 CIN samples ranging from grades I to III, and 19 normal controls, we first employed qRT‐PCR. The melting curve and agarose gel electrophoresis pattern showing TUG1 primer specificity are presented in Figure S1. We observed that TUG1 expression was significantly higher in cervical cancer tissues than in CIN samples (*P *<* *0.001) and normal tissues (*P* < 0.001; Fig. 1A). Furthermore, expression was higher in the CIN samples than in normal tissues (*P *<* *0.01; Fig. 1A). A search of the Gene Expression Omnibus (GEO) database (http://www.ncbi.nlm.nih.gov/gds/) using the Oncomine Platform (https://www.oncomine.org/resource/login.html) revealed that TUG1 expression increased gradually during tumorigenesis from normal tissue to CIN and subsequently, to cervical cancer (Fig. S2). Compared with normal samples, the level of TUG1 was significantly upregulated in all four cervical cancer cell lines, including HPV16(+) SiHa and CaSki cells, HPV18(+) HeLa cells, and HPV(−) C33A cells (Fig. 1B).

### TUG1 overexpression is correlated with the clinicopathological features of cervical cancer patients

Next, we explored the correlation between TUG1 expression and the clinicopathological characteristics of 40 cervical cancer patients (Table 1). Clinical data showed that high TUG1 expression was significantly correlated with larger tumor size (*P *<* *0.001), advanced FIGO stage (*P *=* *0.009), poor differentiation (*P *=* *0.007), and lymph node metastasis (*P *=* *0.015), but not with other clinicopathological factors, such as age, histologic type, and the level of serum squamous cell carcinoma antigen (SCC‐Ag; *P *>* *0.05).

**Table 1:** Correlation between TUG1 expression and clinicopathological features in 40 cervical cancer samples

Clinicopathologic variables		*n*	TUG1^a^	*P* value^b^
Age	≤geueiab	17	0.042 (0.020~0.091)	*P *=* *0.533
	>50 years	23	0.0471 (0.022~0.105)	
Tumor size	≤4 cm	18	0.024 (0.011~0.047)	*P *<* *0.001
	>4 cm	22	0.073 (0.043~0.134)	
FIGO stage	I~IIa	30	0.079 (0.015~0.105)	*P *=* *0.009
	IIb~III	10	0.098 (0.061~0.201)	
Differentiation	Well or moderately	21	0.031 (0.012~0.071)	*P *=* *0.007
	Poorly	19	0.073 (0.046~0.108)	
Histology	Squamous	35	0.046 (0.022~0.103)	*P *=* *1
	Adenocarcinoma	5	0.046 (0.021~0.129)	
SCC‐Ag (*μ*g/L)	≤4 (*μ*g/L)	25	0.042 (0.015~0.070)	*P *=* *0.083
	>4 (*μ*g/L)	15	0.088 (0.028~0.120)	
Lymph node metastasis	Negative	25	0.034 (0.015~0.072)	*P *=* *0.015
	Positive	15	0.088 (0.047~0.120)	

FIGO, international federation of gynecology and obstetrics.

a Median of relative expression, with 25th–75th percentile in parentheses.

b *P* value was calculated by Mann–Whitney U test.

### TUG1 promotes the proliferation of cervical cancer cells

**Figure 2:**
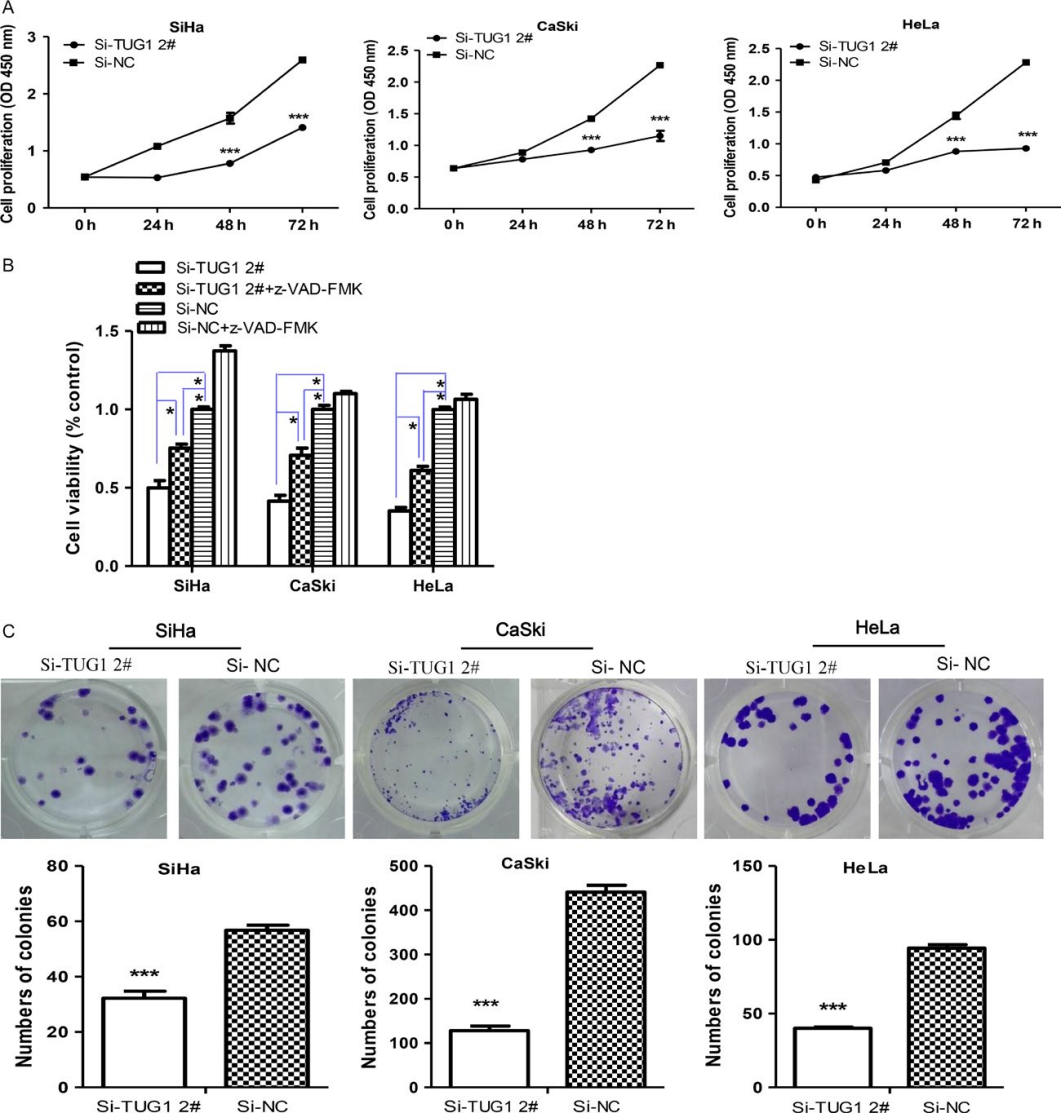
Knockdown of TUG1 inhibits the proliferation and colonization abilities of cervical cancer cells. (A) The Cell Counting Kit‐8 (CCK‐8) assay was used to determine the cell viability. Compared with cells transfected with si‐NC, knockdown of TUG1 significantly inhibited cervical cancer cell proliferation. (B) SiHa, CaSki, and HeLa cells were transfected with si‐TUG1 2# with or without 50 *μ*mol/L z‐VAD‐FMK (ZVAD). Cell proliferation was determined by the CCK‐8 assay at 72 h posttransfection. The effect of si‐TUG1 2# was partly reversed by the broad‐spectrum caspase inhibitor ZVAD. (C) A colony‐forming assay was performed on cervical cancer cells transfected with si‐TUG1 2#. All experiments were performed three times. Data are presented as means ± SEM. *P <* *0.05, ***P <* *0.001.

TUG1 was overexpressed in cervical cancer, allowing us to investigate its biological function upon selective inhibition. We achieved this by transiently transfecting cervical cancer cells with siTUG1 to knockdown TUG1. The suppression efficiency in cells transfected with TUG1 siRNAs (siTUG1 1# and siTUG1 2#) is shown in Figure S3. As indicated by the results of the CCK‐8 assay, transfection of all four cervical cancer cells (SiHa, CaSki, HeLa, and C33A) with siTUG1 2# decreased cell viability by 50.3%, 34.8%, 39%, and 41.8%, respectively (Fig. 2A, Fig. S4A), and siTUG1 1# showed similar results (Fig. S5A). Moreover, the inhibitory effect of siTUG1 on the growth of cervical cancer cell lines occurred in a time‐dependent manner (Fig. 2A, Fig. S4A and Fig. S5B). Similarly, the colony‐forming assay showed that clonogenic survival decreased significantly when cervical cancer cells were transfected with TUG1 siRNAs (siTUG1 1# and siTUG1 2#) (Fig. 2C and Fig. S5C), indicating a proliferation‐promoting function of TUG1 in cervical cancer cells.

### TUG1 knockdown induces apoptosis of cervical cancer cells in association with changes in apoptosis‐related proteins

**Figure 3:**
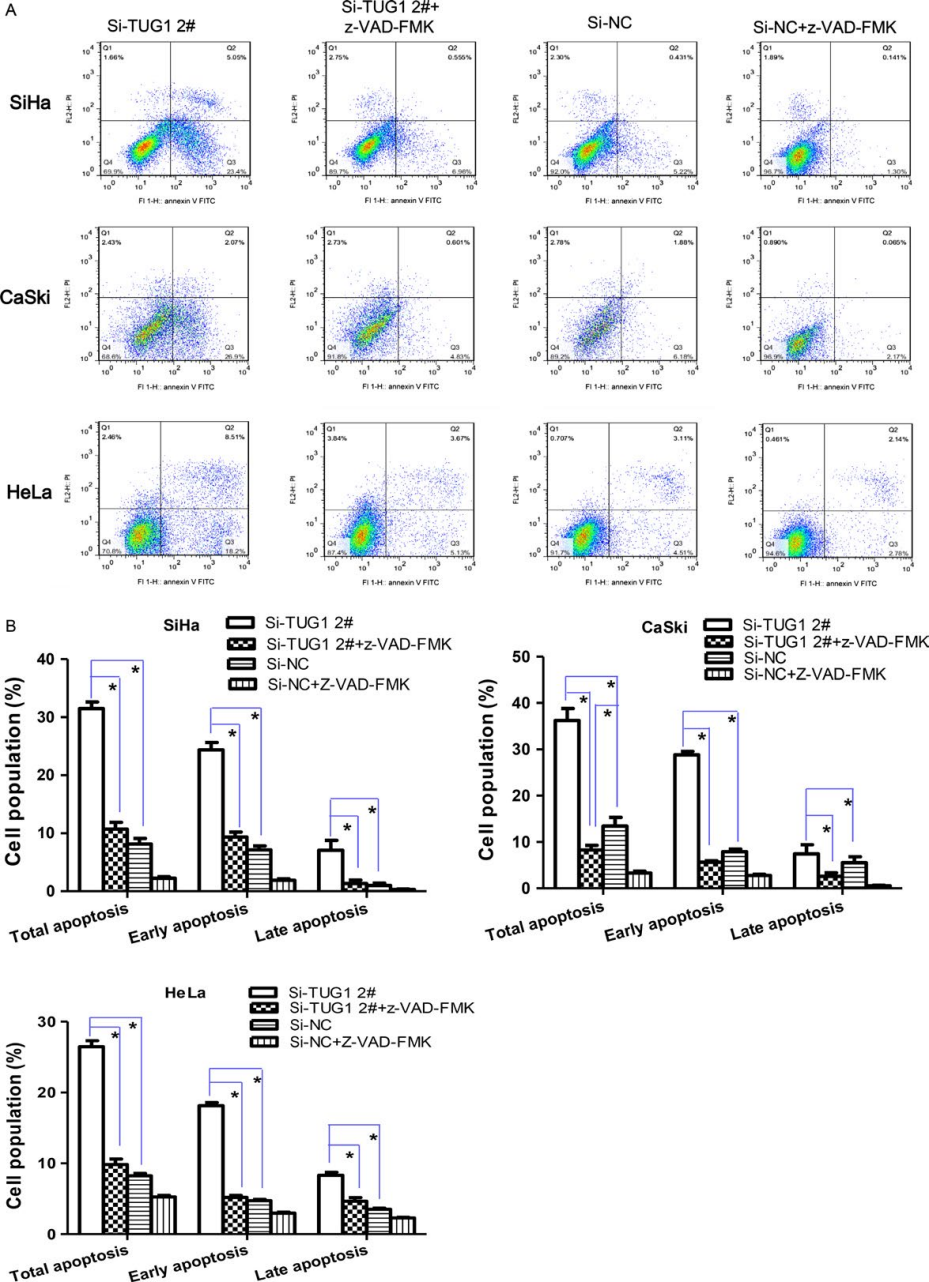
Apoptosis increases in cells transfected with si‐TUG1. (A) Cell apoptosis following transfection for 48 h with si‐TUG1 2# or si‐NC was measured by flow cytometry with or without z‐VAD‐FMK treatment. (B) Percentage of total, early, and late apoptotic cervical cancer cells. Data are presented as means ± SEM of three independent experiments. *P <* *0.05.

To investigate whether the effect of TUG1 on the proliferation of cervical cancer cells involved changes in apoptosis, cells were transiently transfected with siTUG1 for 48 h, and then stained with Annexin V/PI. The extent of apoptosis was then measured by flow cytometry. Our results showed an elevated number of Annexin V+ (total apoptosis), Annexin V+/PI‐ (early apoptosis), and Annexin V+/PI+ (late apoptosis) cells after TUG1 knockdown (Fig. 3A, Fig. S4B, and Fig. S6A). The percentage of apoptotic cells at each stage was markedly higher in siTUG1‐treated cells than in the negative control siRNA group (Fig. 3B and Fig. S4C); however, this effect could be prevented by treatment with ZVAD, a broad‐spectrum irreversible caspase inhibitor (Fig. 3B, Fig. S4C, and Fig. S6B). Accordingly, the percentage of both early and late apoptotic SiHa, CaSki, HeLa, and C33A cells decreased significantly after treatment with ZVAD for 48 h. At the same time, exposure to ZVAD prevented the death of SiHa, CaSki, and HeLa cells caused by TUG1 knockdown, as measured by CCK‐8 assays (Fig. 2B, Fig. S5B).

**Figure 4:**
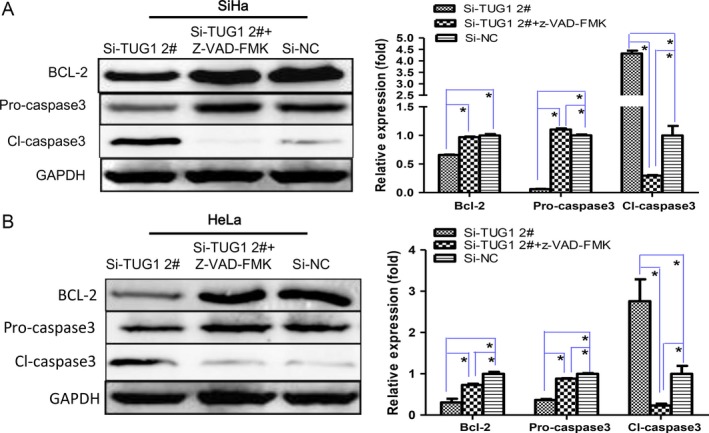
TUG1 affects the expression of apoptosis‐related proteins. The expression of Bcl‐2, procaspase‐3, and cleaved caspase‐3 was determined by Western blotting in (A) SiHa and (B) HeLa cells transfected with si‐TUG1 2# with or without 50 *μ*mol/L z‐VAD‐FMK treatment. GAPDH was used as the internal reference. Pixel densities were analyzed and are presented as histograms. Data are presented as means ± SEM of three independent experiments. *P <* *0.05.

Furthermore, the level of Bcl‐2 protein was greatly decreased upon TUG1 knockdown (Fig. S7). Additionally, we observed a significant increase in caspase‐3 activation. Specifically, the expression of cleaved caspase‐3 increased, whereas that of precursor caspase‐3 decreased significantly (Fig. S7). As expected, the effect of TUG1 knockdown on these apoptotic proteins was partially prevented by treatment with ZVAD (Fig. 4).

### Knockdown of TUG1 decreases migration and invasion of cervical cancer cells

**Figure 5:**
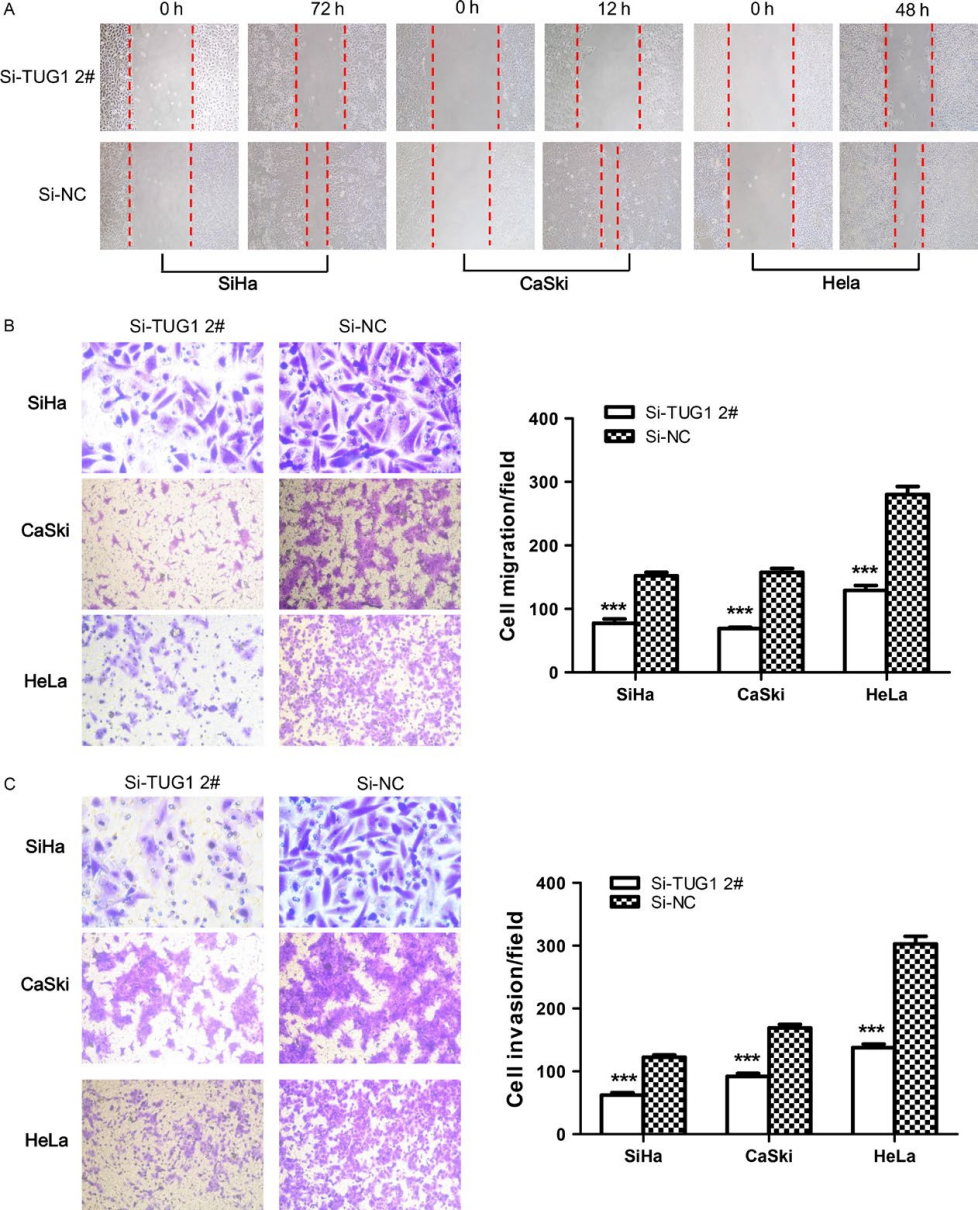
Knockdown of TUG1 restrains the migration and invasion of cervical cancer cells. (A) The motility of cervical cancer cells transfected with si‐TUG1 2# decreased compared with cells treated with si‐NC, as detected by cell wound healing assay. Images (100× magnification) were taken at the indicated times after wounding at the same location in each well. Knockdown of TUG1 decreased (B) migration and (C) invasion of cervical cancer cells, as detected by migration and invasion transwell assays. The number of invading cells was counted by analyzing photographs at 100× magnification in five random fields per chamber. Data are expressed as means ± SEM of three independent experiments. ***P <* *0.001.

To investigate the involvement of TUG1 in cell migration and invasion, we performed a cell wound healing assay. Cells treated with siTUG1 exhibited a lower degree of wound closure than the control group (Fig. 5A, Fig. S8A). Additionally, we utilized a classical transwell assay to assess the effect of TUG1 on the migration and invasion of SiHa, CaSki, and HeLa cells. The results showed a notable decrease in the number of migrated and invaded cells transfected with siTUG1 relative to the negative control siRNA group (Fig. 5B,C and Fig.S8B,C). These results indicated that TUG1 promoted migration and invasion of cervical cancer cells.

### TUG1 knockdown suppresses the expression of EMT‐related proteins

**Figure 6:**
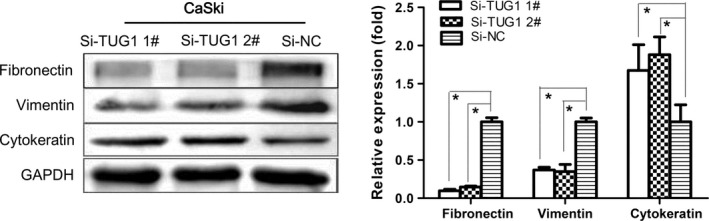
TUG1 knockdown suppresses the expression of epithelial‐to‐mesenchymal transition (EMT)‐related proteins. Changes in fibronectin, vimentin, and cytokeratin protein levels were determined by Western blotting. Data are expressed as means ± SEM of three independent experiments. *P <* *0.05.

We further performed in vitro tests to assess whether the mechanism of action of TUG1 involved effects on EMT. Specifically, we examined fluctuations of EMT‐related markers, fibronectin, vimentin, and cytokeratin, following transfection of CaSki cells, a small bowel mesentery metastatic cervical cancer cell line, with siTUG1. Western blotting confirmed that protein expression of the mesenchymal markers, fibronectin and vimentin, was significantly inhibited in CaSki cells with siTUG1 interference compared with control cells, whereas the opposite effect was observed with respect to the expression of cytokeratin, an epithelial marker (Fig. 6).

## Discussion

A large number of studies have shown that mammalian genomes encode thousands of lncRNAs 6. Accordingly, it has become increasingly clear that lncRNA dysregulation, linked to cancers with distinct modes of action, is involved in the tumorigenesis and progression of malignant tumors 7, 8, 26. TUG1 is anomalously expressed in several human cancers. However, specific changes in TUG1 expression vary greatly in different cancers 19, 20, 21, 22, 23, 27, 28. In this study, we demonstrated that TUG1 expression was significantly higher in cervical cancer specimens and cervical cancer cell lines than in normal tissues. In addition, we found that the expression of TUG1 in CIN was higher than that in normal cervical uterus tissue, but lower than that in cervical cancer. This finding was consistent with the level of TUG1 expression reported in several other solid tumors 22, 23, 28. Moreover, published data in the GEO database confirmed that TUG1 is significantly upregulated in cervical cancer compared with normal tissues, including the cervix uteri, oral cavity, palate, and tonsils. However, a previous study has demonstrated that TUG1 is downregulated in nonsmall cell lung cancer 19, which is probably because lncRNAs exhibit remarkably tissue‐specific expression patterns, in contrast to protein‐coding genes 29. Analysis of the clinicopathological characteristics of patients with cervical cancer revealed that TUG1 upregulation was correlated with larger tumor size, advanced FIGO stage, lower differentiation, and lymph node metastasis. Similarly, a high expression level of TUG1 has been found to be associated with the degree of malignancy in several other tumors 21, 22. These results indicate that TUG1 expression is associated with the level of malignancy of cervical cancer, and that it participates in the tumorigenesis and progression of this disease.

Several reports have revealed that the effect of TUG1 in cancer relates to its biological role in cell proliferation, apoptosis, migration, and metastasis 19, 20. Therefore, we investigated the biological function of TUG1 via RNA interference‐mediated knockdown (loss‐of‐function approach) using different types of cervical cancer cell lines. Downregulation of TUG1 significantly reduced cell growth in cervical cancer cells and induced cell apoptosis. Interestingly, TUG1 was found to influence primarily early apoptosis in HPV(+) cells, including HPV16(+) SiHa and CaSki cells, and HPV18(+) HeLa cells, but mainly late apoptosis in HPV(−) C33A cells. The exact mechanism by which TUG1 knockdown influences different stages of apoptosis in HPV(+) and HPV(−) cells requires further investigation.

A growing number of studies have demonstrated that lncRNAs exert their biological functions by binding to important downstream proteins 30. Bcl‐2 protein is a potent tumor suppressor and an important player in apoptotic signaling. Bcl‐2 blocks the increased permeability of the mitochondrial membrane and prevents the release of cytochrome C 31. Caspase‐3, the principal end‐cleaving protein in programmed cell death, plays a crucial role in dismantling the cell, leading to the formation of apoptotic bodies 32; this effect may be blocked by Bcl‐2 33. Here, we show that TUG1 downregulation decreased Bcl‐2 protein levels and induced caspase‐3 cleavage. In addition, ZVAD prevented changes in apoptosis‐related proteins and suppressed apoptosis in cells treated with siTUG1. This indicates that TUG1 acts as a tumor promoter in cervical cancer by increasing cell proliferation and decreasing cell apoptosis by upregulating Bcl‐2 expression and suppressing the activation of caspase‐3. However, the exact reason for this change remains to be further studied.

Our results demonstrated that TUG1 affects the motility of cervical cancer cells. Cell wound healing and transwell assays suggested that TUG1 knockdown inhibits the migratory and invasive ability of cervical cancer cells. To investigate the mechanism underlying the migration process, we measured the protein level of EMT markers following ectopic expression of siTUG1. EMT endows cells with migratory and invasive properties 34, making it one of the most crucial steps in tumor metastatic progression. EMT controls the intercellular adhesion mechanism and cytoskeletal dynamics 35. Furthermore, EMT plays a transient role in early tumor invasion and metastasis via several molecular pathways, enabling blood or lymph vessel intravasation 36. Our results suggest that TUG1 knockdown activates cytokeratin expression and decreases the expression of vimentin and fibronectin in cervical cancer, partly explaining the metastasis‐related mechanism in this disease.

In conclusion, to our knowledge, this study is the first to show that lncRNA‐TUG1 participates in the progression of cervical cancer. TUG1 expression is upregulated in cervical cancer, where it correlates with larger tumor size, advanced FIGO stage, lower differentiation, and lymph node metastasis. Knockdown of TUG1 exerts tumor‐suppressive functions by reducing cell proliferation and inducing cell apoptosis via the caspase signaling pathway. In addition, we clarified that TUG1 promotes migration and invasion of cervical cancer cells, in part by regulating EMT. The present findings increase our understanding of the pathogenesis of cervical cancer and implicate TUG1 as a potential new target for the treatment of this disease.

## Conflict of Interest

The authors declare no conflicts of interest.

## Supporting information


**Figure S1.** Specificity of TUG1 primers in qRT‐PCR assays. (A) Agarose gel electrophoresis of reactions amplified with TUG1 primers over a temperature gradient. Melting curves for (B) TUG1 and (C) GAPDH. (D) Sequencing results of the TUG1 PCR product.

**Figure S2.** Genome‐wide analysis of TUG1 expression in Gene Expression Omnibus (GEO) datasets. (A) Relative expression of TUG1 in cervical cancer compared with normal tissues was analyzed with Oncomine platform based on the GEO database. 1, Cervix uteri (*n* = 8); 2,Oral cavity (*n* = 9); 3, Palate (*n* = 1); 4,Tonsil (*n* = 4); 5. Cervical cancer (*n* = 20). (B) TUG1 expression in cervical cancer was compared with that in high‐grade cervical squamous intraepithelial neoplasia (HSIL) and low‐grade cervical squamous intraepithelial neoplasia (LSIL) using the Oncomine platform based on the GEO database. 0. Cervical Squamous Cell Carcinoma (*n* = 10); 1, HSIL (*n* = 24); 2, LSIL (*n* = 7).

**Figure S3.** Suppression efficiency of si‐TUG1(si‐TUG1 1#, 2#) in CaSki cells. The relative expression of TUG1 in CaSki cells transfected with si‐TUG1 or si‐NC was assessed by qRT‐PCR. Data are presented as means ± SEM of three independent experiments. **P <* *0.01, ***P <* *0.001.

**Figure S4.** TUG1 depletion inhibits cell proliferation and promotes cell apoptosis in C33A cells. (A) The CCK‐8 assay showed that the growth rate of C33A cells was suppressed in groups transfected with si‐TUG1 (si‐TUG1 1#, 2#). (B) C33A cells were transfected with si‐TUG1, treated with or without 50 *μ*mol/L z‐VAD‐FMK, and analyzed by flow cytometry at 48 h posttransfection. (C) Relative percentage of total, early, and late apoptotic C33A cells. Data are presented as means ± SEM of three independent experiments. *P <* *0.05, ***P <* *0.001.

**Figure S5.** Impact of si‐TUG1 1# on the proliferation and colony‐forming abilities of cervical cancer cells. (A) The CCK‐8 assay was used to assess the proliferation of cervical cancer cells 48 h after transfection with si‐TUG11# or si‐NC. (B) The proliferation inhibitory effect of si‐TUG1 1# could be partly reversed by z‐VAD‐FMK treatment. (C) A colony‐forming assay was performed on si‐TUG1 1#‐transfected cervical cancer cells. All experiments were performed in triplicate. **P <* *0.01, ***P <* *0.001.

**Figure S6.** Knockdown of TUG1 accelerates cell apoptosis in cervical cancer cells. (A) Cell apoptosis following transfection for 48 h with si‐TUG1 1# was measured by flow cytometry with or without z‐VAD‐FMK treatment. (B) Percentage of total, early, and late apoptotic cervical cancer cells. Data are presented as means ± SEM of three independent experiments. *P <* *0.05.

**Figure S7.** Changes in apoptosis‐related proteins in transfected cervical cancer cells. Changes in the expression of apoptosis‐related proteins in (A) SiHa and (B) HeLa cells were determined by western blotting at 48 h post‐transfection. GAPDH served as a loading control. *P <* *0.05.

**Figure S8.** Transfection of si‐TUG1 1# suppresses the migration and invasion abilities of cervical cancer cells. (A) Motility of cervical cancer cells transfected with si‐TUG1 1# decreased compared with cells treated with si‐NC, as detected by cell wound healing assay. Knockdown of TUG1 reduced (B) migration and (C) invasion of cervical cancer cells, as detected by migration and invasion transwell assays. Representative images of the migration and invasion transwell assay are shown. Data are expressed as means ± SEM of three independent experiments. **P <* *0.01, ***P <* *0.001.
