# Supplementary figures and images for: Upregulation of long noncoding RNA TUG1 promotes cervical cancer cell proliferation and migration
Source: Cancer Med. 2017 Jan 15;6(2):471–82. doi: 10.1002/cam4.994 (PMC5313648; doi:10.1002/cam4.994)

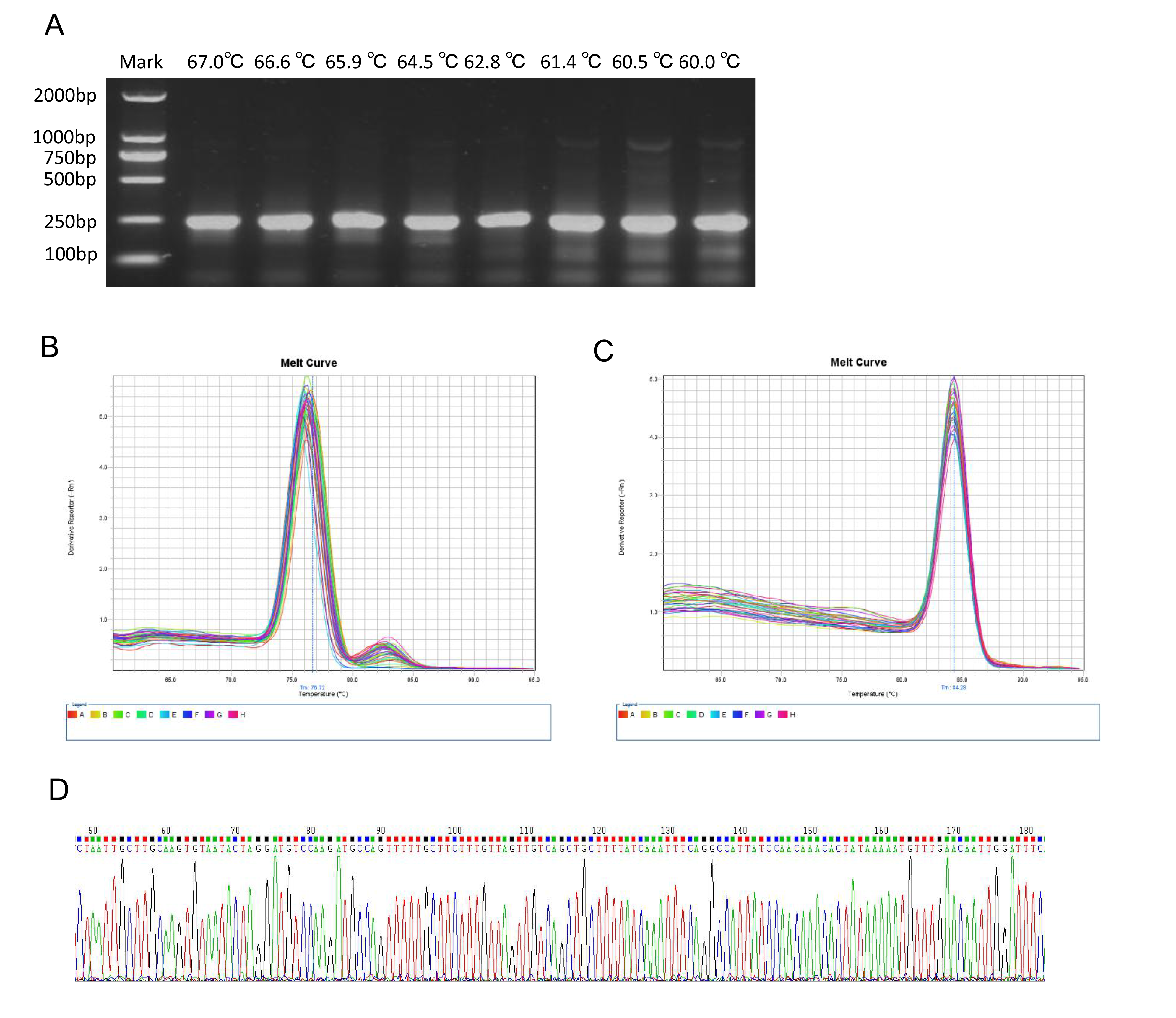

Supplement: Supplementary file 1 — Figure S1. Specificity of TUG1 primers in qRT‐PCR assays. (A) Agarose gel electrophoresis of reactions amplified with TUG1 primers over a temperature gradient. Melting curves for (B) TUG1 and (C) GAPDH. (D) Sequencing results of the TUG1 PCR product. [file CAM4-6-471-s001.tif]

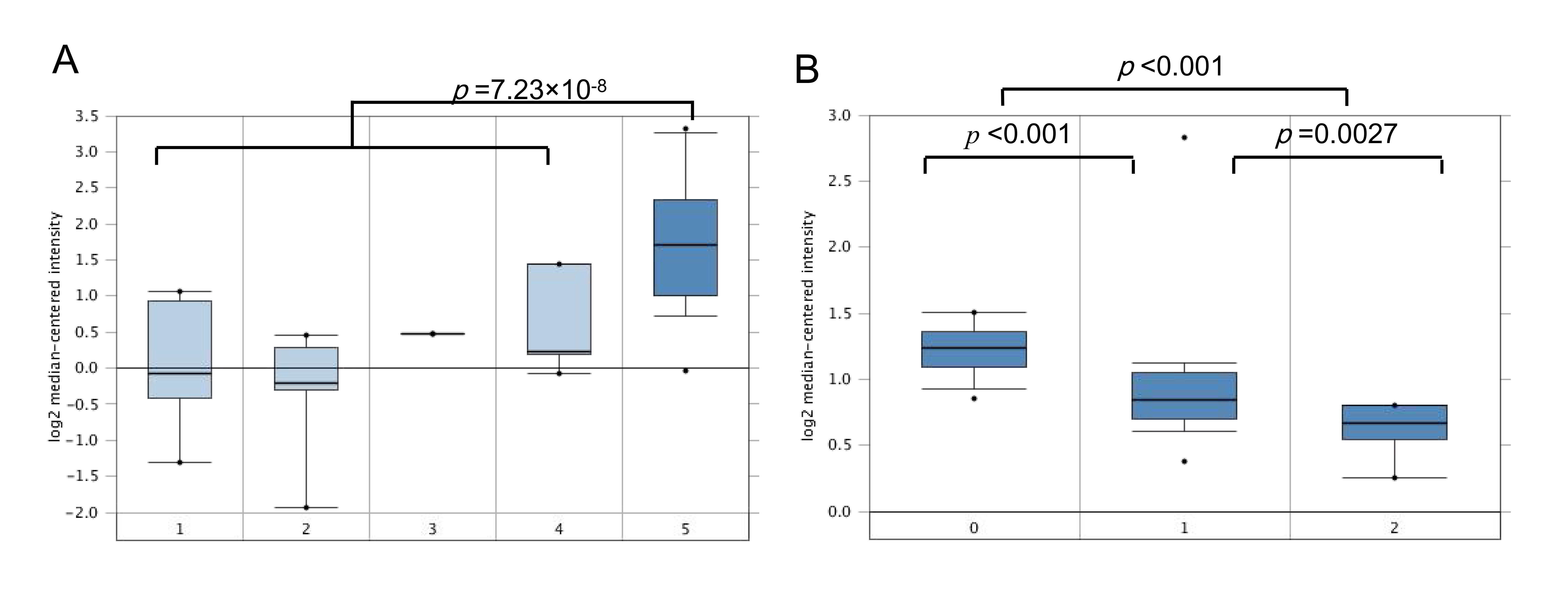

Supplement: Supplementary file 2 — Figure S2. Genome‐wide analysis of TUG1 expression in Gene Expression Omnibus (GEO) datasets. (A) Relative expression of TUG1 in cervical cancer compared with normal tissues was analyzed with Oncomine platform based on the GEO database. 1, Cervix uteri (n = 8); 2,Oral cavity (n = 9); 3, Palate (n = 1); 4,Tonsil (n = 4); 5. Cervical cancer (n = 20). (B) TUG1 expression in cervical cancer was compared with that in high‐grade cervical squamous intraepithelial neoplasia (HSIL) and low‐grade cervical squamous intraepithelial neoplasia (LSIL) using the Oncomine platform based on the GEO database. 0. Cervical Squamous Cell Carcinoma (n = 10); 1, HSIL (n = 24); 2, LSIL (n = 7). [file CAM4-6-471-s002.tif]

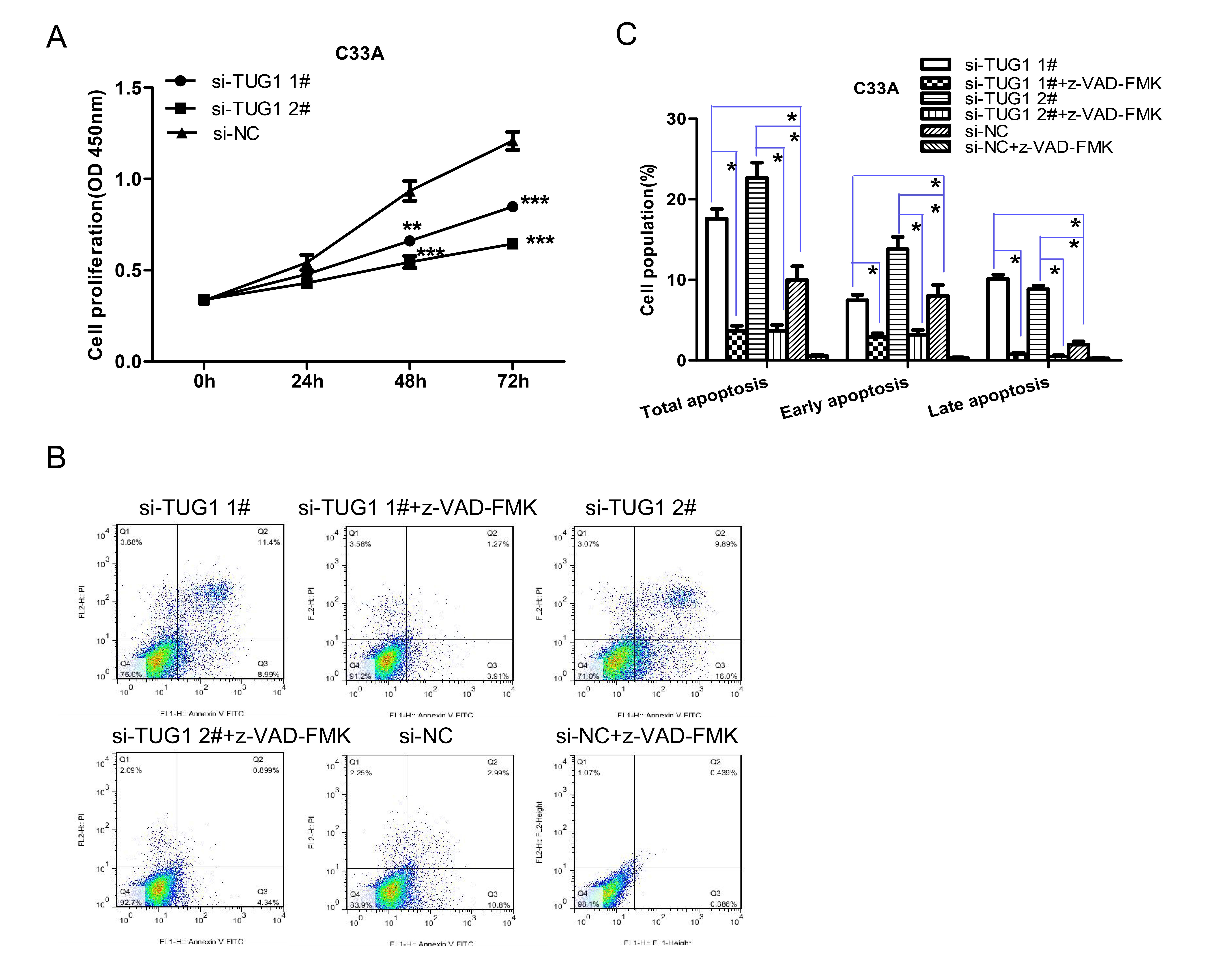

Supplement: Supplementary file 4 — Figure S4. TUG1 depletion inhibits cell proliferation and promotes cell apoptosis in C33A cells. (A) The CCK‐8 assay showed that the growth rate of C33A cells was suppressed in groups transfected with si‐TUG1 (si‐TUG1 1#, 2#). (B) C33A cells were transfected with si‐TUG1, treated with or without 50 μmol/L z‐VAD‐FMK, and analyzed by flow cytometry at 48 h posttransfection. (C) Relative percentage of total, early, and late apoptotic C33A cells. Data are presented as means ± SEM of three independent experiments. *P < 0.05, ***P < 0.001. [file CAM4-6-471-s004.tif]

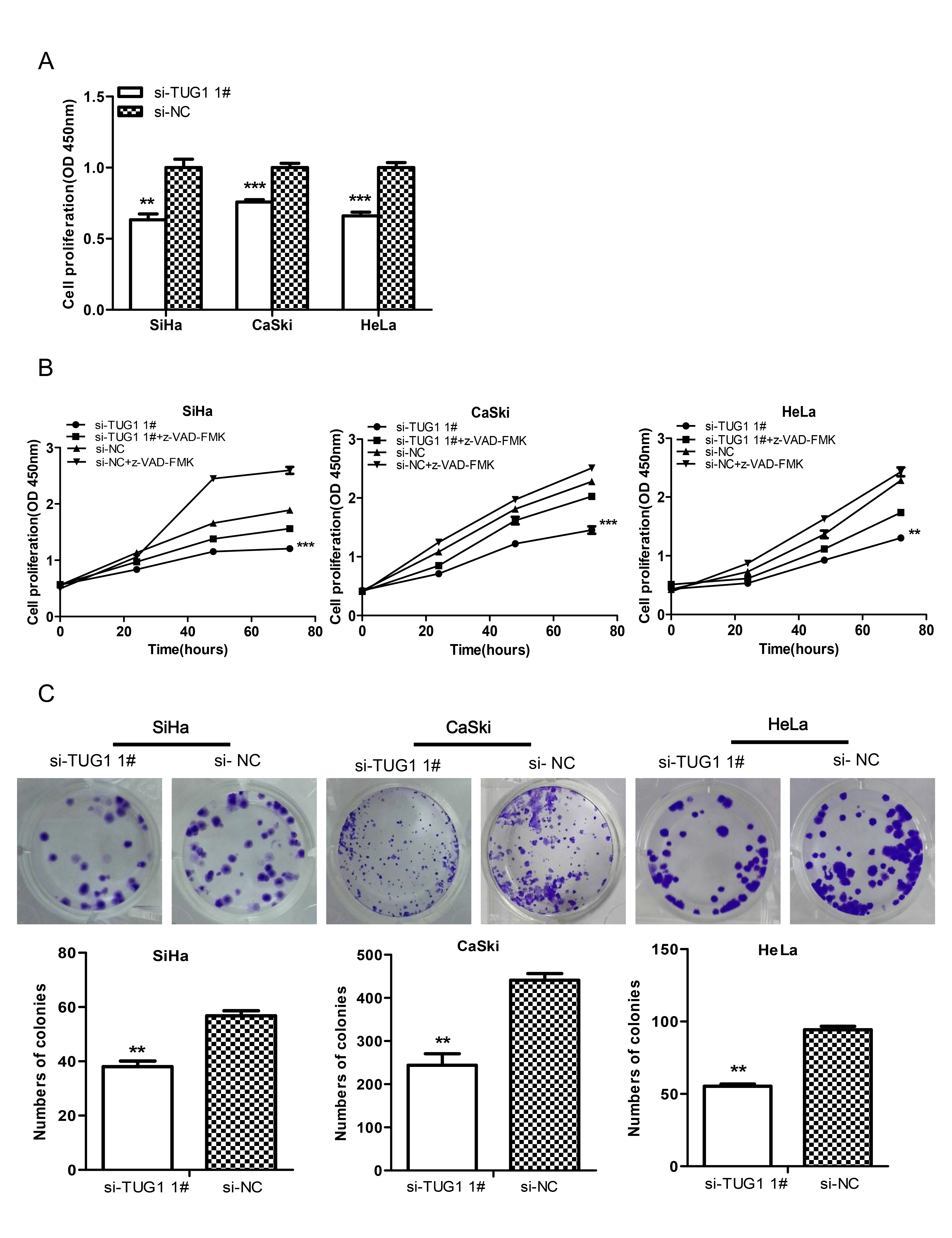

Supplement: Supplementary file 5 — Figure S5. Impact of si‐TUG1 1# on the proliferation and colony‐forming abilities of cervical cancer cells. (A) The CCK‐8 assay was used to assess the proliferation of cervical cancer cells 48 h after transfection with si‐TUG11# or si‐NC. (B) The proliferation inhibitory effect of si‐TUG1 1# could be partly reversed by z‐VAD‐FMK treatment. (C) A colony‐forming assay was performed on si‐TUG1 1#‐transfected cervical cancer cells. All experiments were performed in triplicate. **P < 0.01, ***P < 0.001. [file CAM4-6-471-s005.tif]

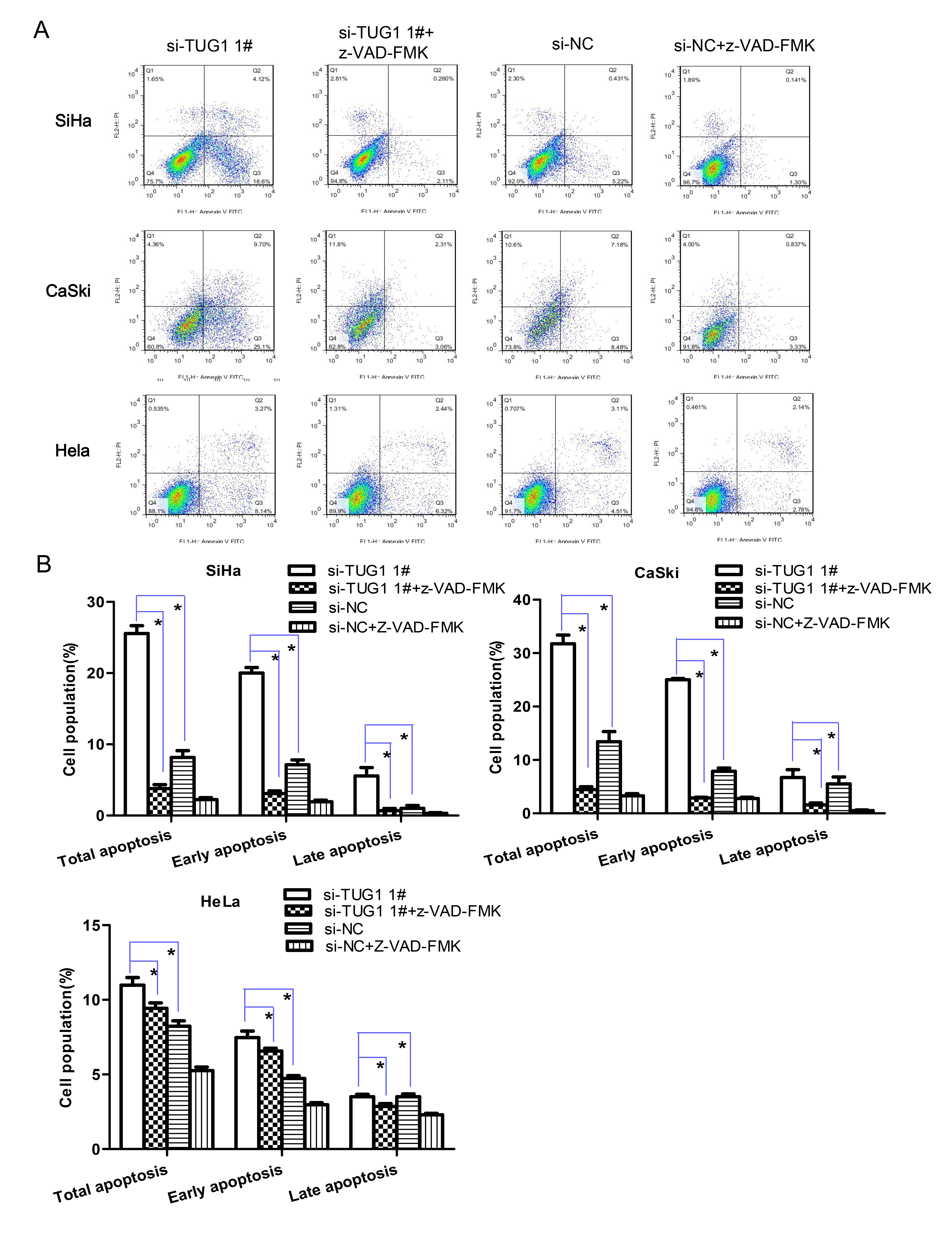

Supplement: Supplementary file 6 — Figure S6. Knockdown of TUG1 accelerates cell apoptosis in cervical cancer cells. (A) Cell apoptosis following transfection for 48 h with si‐TUG1 1# was measured by flow cytometry with or without z‐VAD‐FMK treatment. (B) Percentage of total, early, and late apoptotic cervical cancer cells. Data are presented as means ± SEM of three independent experiments. *P < 0.05. [file CAM4-6-471-s006.tif]

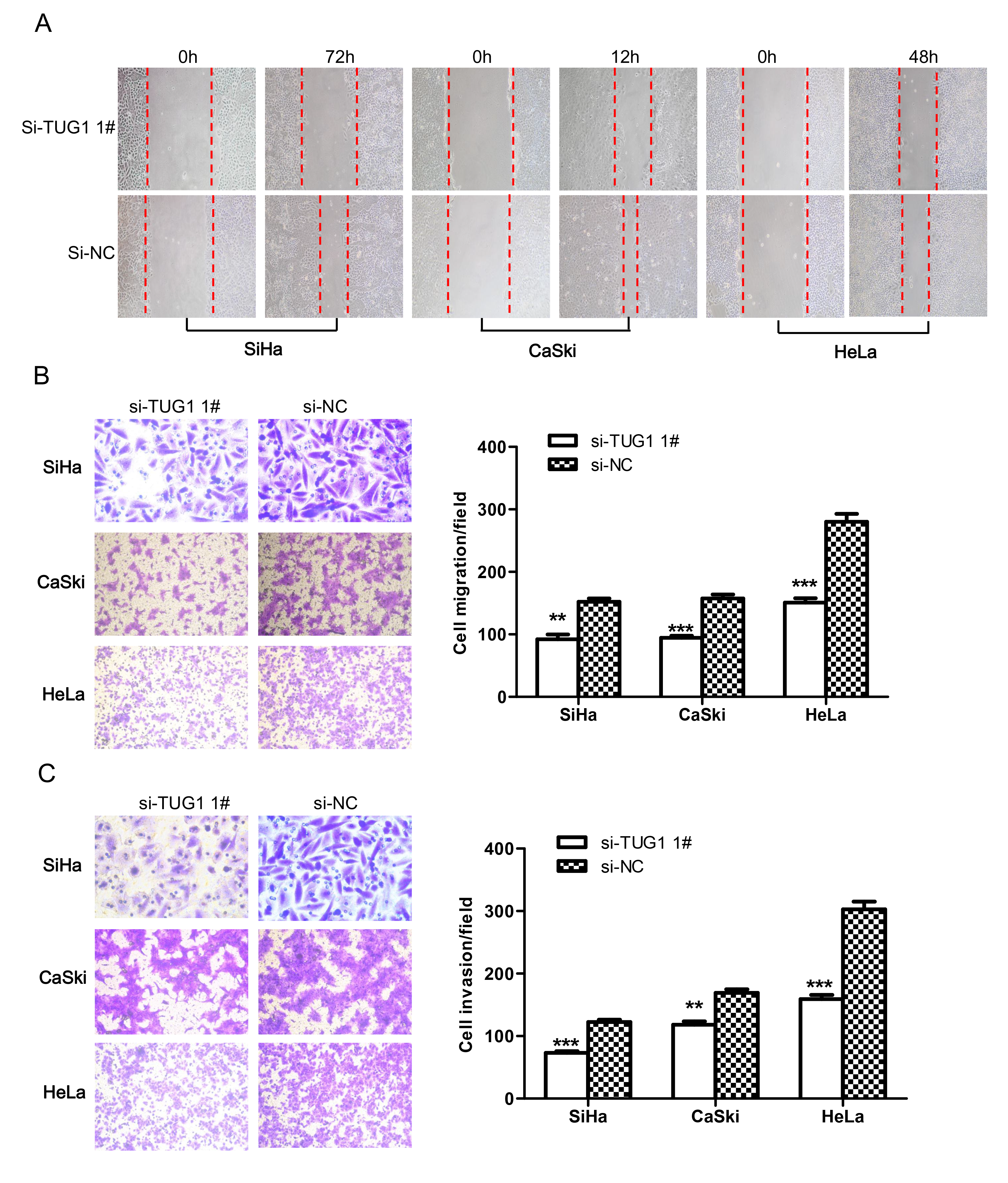

Supplement: Supplementary file 8 — Figure S8. Transfection of si‐TUG1 1# suppresses the migration and invasion abilities of cervical cancer cells. (A) Motility of cervical cancer cells transfected with si‐TUG1 1# decreased compared with cells treated with si‐NC, as detected by cell wound healing assay. Knockdown of TUG1 reduced (B) migration and (C) invasion of cervical cancer cells, as detected by migration and invasion transwell assays. Representative images of the migration and invasion transwell assay are shown. Data are expressed as means ± SEM of three independent experiments. **P < 0.01, ***P < 0.001. [file CAM4-6-471-s008.tif]
